# Improving distributed PV integration with dynamic thermal rating of power distribution equipment

**DOI:** 10.1016/j.isci.2022.104808

**Published:** 2022-07-21

**Authors:** Yinxiao Li, Yi Wang, Chongqing Kang, Jie Song, Guannan He, Qixin Chen

**Affiliations:** 1 Department of Electrical Engineering, Tsinghua University, Beijing 100084, China; 2 Department of Electrical and Electronic Engineering, The University of Hong Kong, Hong Kong, China; 3 Department of Industrial Engineering and Management, College of Engineering, Peking University, Beijing 100871, China; 4 MIT Energy Initiative, Massachusetts Institute of Technology, Cambridge, MA 02139, USA

**Keywords:** Applied sciences, Engineering, Power engineering

## Abstract

The rapid development of distributed photovoltaic (PV) systems poses great challenges to the integration capability of distribution networks. Traditionally, the transfer capacity of power distribution equipment is calculated as the maximum loading that prevents overheating under the assumption of extreme weather conditions. Dynamic thermal rating (DTR), which evaluates equipment capacity based on real-time weather conditions, could enhance the transfer capacity to improve distributed PV integration. Through case studies in Texas, Switzerland, and China, we show that the application of DTR on power distribution equipment could increase installed PV capacities by 15%–27% and improve net revenues by 4%–27%. We also find that the application of DTR would be positively affected by climate change and is more profitable under the PV policies with higher tariffs for the surplus generation fed into the grid. Compared to energy storage systems, DTR provides a more cost-competitive option to enhance the integration capability of distribution networks.

## Highlights

• Dynamic thermal rating improves PV integration capability of distribution networks
• The improving effects would be positively affected by climate change
• Dynamic thermal rating is more profitable with high tariffs for feed-in PV generation
• Dynamic thermal rating is more cost-competitive than energy storage systems

## Graphical abstract

**Figure:**
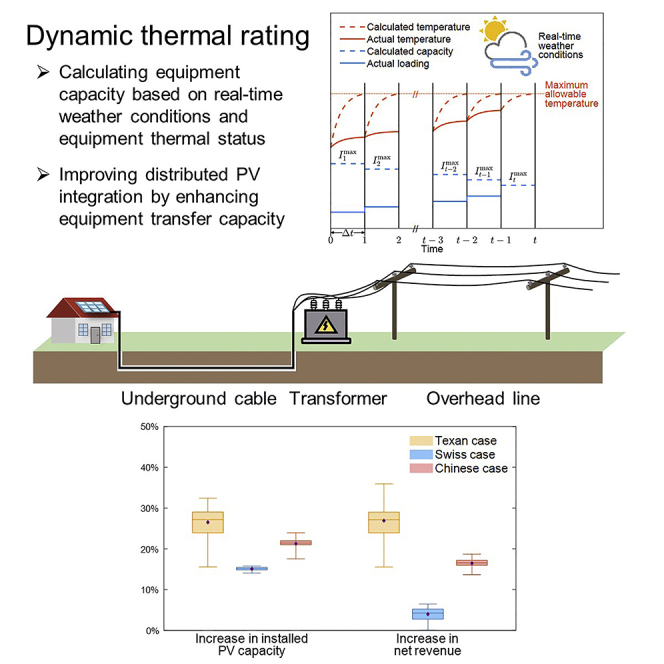

## Introduction

Solar photovoltaic (PV) resources have been developing rapidly around the world and will play a critical role in supporting energy transition (IEA 2021). Because of its capability to meet local energy demand without heavy investments and power losses of long-distance power transmission (Jain et al., 2017; Sutherland 2018), there has been an increasing interest in distributed PV resources, the installed capacity of which has accounted for approximately 40% of total PV capacity in recent years (IEA 2020d). Moreover, relevant studies show that the potential capacities of rooftop PV in the European Union, the United States, China, and worldwide are estimated at 951 GW, 1,118 GW, 1,150 GW, and 19,385 GW, respectively (Defaix et al., 2012; Gagnon et al., 2016; Wang et al., 2021; Joshi et al., 2021), indicating the considerable potential for distributed PV development.

Traditional power distribution networks were planned only to meet local power loads, with power flowing from substations toward end customers (Koutsoukis et al., 2018). If the generation from distributed PV systems exceeds the power load, the direction of power flow will be reversed. With high distributed PV penetration, large reverse power flows could become more common and reach the distribution network capacity limit designed for peak load, leading to bottlenecks for further PV integration (Alam et al., 2013). For example, the PV capacity exceeds the peak load by 900% in a real distribution network in southern Germany (Appen et al., 2013), and the penetration rate of distributed renewable energy (ratio of installed renewable energy capacity to peak load) reaches 310% and 216% in two demonstration projects in China (Sheng et al., 2019). The large reverse power flows might cause operating problems during midday PV generation peaks and compromise grid reliability, such as equipment overloading and grid overvoltage (Horowitz et al., 2020). Without significant upgrades, it would be difficult for distribution networks to support the rapid and sustainable development of distributed 10.13039/501100013891PV resources (Fares and Webber 2017). For example, distribution networks with different voltage levels must expand by 5%–14% to support the distributed power integration necessary to achieve Germany’s goal of 50% renewable energy penetration by 2032 (Büchner et al., 2015). In addition to traditional grid upgrades (e.g., reconductoring or replacement of the equipment), existing studies have explored low-cost technologies to improve the integration capability and hosting capacity of distribution networks by addressing the grid overvoltage problem, such as advanced inverter control (Horowitz et al., 2020), on-load tap changer of transformers (Ku et al., 2019), and reactive power compensation (Long and Ochoa 2015). However, these technologies can only improve the integration capability and hosting capacity without overloading the equipment. When the distribution networks are facing the overloading problem of transformers and lines, further increases in distributed PV penetration would require significant upgrade costs, as the traditional grid upgrades are generally expensive (Horowitz et al., 2018). Therefore, insufficient equipment capacity remains an ongoing challenge and has become a bottleneck in distributed PV integration.

In addition to traditional grid upgrades, dynamic thermal rating (DTR) is an effective technology that can enhance the transfer capacity of existing equipment without incurring massive investment costs and time-consuming construction processes (Yang et al., 2015). The nameplate capacity of power equipment is traditionally set as the static thermal rating (STR) and calculated as the maximum constant loading to ensure that the temperatures of key equipment components do not exceed upper limits under the most extreme weather conditions (IEEE 2013, 2012; IEC 2014). In contrast, the DTR of equipment is calculated according to the real-time weather conditions and actual equipment temperatures, which can enhance the transfer capacity of equipment when the weather conditions are mild or only short-term overloading is required (Humayun et al., 2015; Singh et al., 2021). The application of DTR on lines and transformers in the management and dispatch of power systems could address the equipment overloading problem without compromising the operating safety and the service life of the equipment (Bracale et al., 2019). By installing monitoring devices on the equipment and integrating capacity calculation algorithms into energy management systems, DTR can be implemented rapidly and effectively at low costs (IRENA 2020).

Existing literature related to DTR is mainly focused on the application in transmission networks to support centralized renewable energy integration. Relevant studies have explored the optimization methods of transmission network dispatching (Teng et al., 2018; Mohamed et al., 2020) and planning (Zhan et al., 2018; Madadi et al., 2020) with the DTR of overhead lines. The results show that the application of DTR can effectively alleviate congestion, support renewable energy integration, and reduce operating cost and network investment. Viafora et al. (2019) propose a dispatch optimization method incorporating the DTR of both overhead lines and transformers, which can further improve the transfer capacity of the network compared to applying DTR to single-class equipment. At the distribution level, several studies related to distributed renewable energy resources are limited to the optimal dispatch problem of grid operation (Degefa et al., 2014; Safdarian et al., 2015; Li et al., 2021) or analysis of one-node case (Li et al., 2020b). There is a lack of analysis on the benefits of DTR for improving PV planning and investment at the distribution network level, as well as analysis that considers the impacts of climate change and different PV policy scenarios.

In this paper, we comprehensively analyze the improvements of distributed PV integration by the DTR of power distribution equipment. The improvements of distributed PV integration by DTR refer to how much the installed capacities and net revenues of customers’ distributed PV systems can be improved by the application of DTR. The case studies in Texas, Switzerland, and China show that the application of DTR could effectively enhance the transfer capacity and the PV integration capability of distribution networks, thereby increasing the installed PV capacities by 15%–27% and improving the net revenues by 4%–27% on average. Then, we analyze the impacts of climate change and different PV policies on the application of DTR. We find that climate change has positive impacts on the improvements by DTR and DTR is more profitable under the PV policies with higher tariffs for the surplus generation fed into the grid. Furthermore, we also find that DTR is more cost-competitive than deploying energy storage systems (ESSs) to integrate PV systems of the same capacities.

## Results

### Improvements of distributed PV integration by DTR

**Figure 1:**
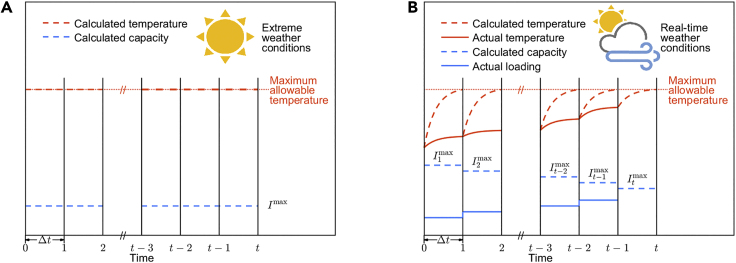
Power distribution equipment capacity rating methods of STR and DTR (A) STR. STR is constant and calculated as the maximum constant loading that prevents overheating under extreme weather conditions, as illustrated by the maximum allowable loading $I^{\text{max}}$ in (A). (B)DTR. DTR is time-varying and weather-related. For each time period (1, 2, …, t−2, t−1, *t*), the capacity is calculated as the highest loading that the equipment can bear without overheating during the time period according to the real-time weather conditions and the initial temperature, as illustrated by the maximum allowable loading $I_{1}^{\text{max}}$, $I_{2}^{\text{max}}$, …, $I_{t-2}^{\text{max}}$, $I_{t-1}^{\text{max}}$, $I_{t}^{\text{max}}$ in (B).

Safe operation of distribution networks requires the loading of power distribution equipment to be kept within reasonable ranges to avoid overheating. The temperatures of key components of power distribution equipment are affected by loading and time-varying weather factors (e.g., ambient temperature, wind, and solar radiation). The greater the loading and the more extreme the weather conditions, the higher the temperatures of the equipment. Traditionally, the capacity of power distribution equipment is set as STR, which is the same for all time periods and is calculated as the maximum constant loading that prevents overheating under extreme weather conditions (for example, assuming high ambient air temperature, high solar radiation, and low wind speed when calculating overhead line capacity), as illustrated in Figure 1A. This method can ensure the safe operation of the equipment at any time without monitoring weather conditions or the thermal status of the equipment.

Nevertheless, STR underestimates the actual transfer capability of the equipment due to the conservative assumption regarding weather conditions. In contrast to STR, DTR treats the equipment capacity as time-varying and evaluates it based on the real-time weather conditions and the actual equipment temperatures to cope with the stochastic and fluctuating characteristics of weather conditions, as shown in Figure 1B. At the beginning of each time period, the DTR is calculated as the highest loading that the equipment can bear without overheating during the time period according to the real-time weather conditions and the initial equipment temperature. Then, keeping the equipment loading below the calculated real-time capacity can prevent equipment overheating and ensure safe operation. The milder the weather conditions and the lower the initial temperature, the higher the equipment capacity.

We use distribution network systems (Li et al., 2020a; Zu et al., 2019) and weather data (Muñoz Sabater 2019) from Texas, Switzerland, and China to analyze the improvements of distributed PV integration by DTR. In the STAR Methods Section, we provide the details of the data. In the case study, we compare and analyze the results of two scenarios applying STR and DTR, named Scenario STR and Scenario DTR. The improvements of distributed PV integration by DTR are analyzed by comparing the installed capacities and net revenues of customers’ distributed PV systems in the two scenarios. During the planning of the distributed PV systems, we assume that each customer in the distribution network first plans PV capacity individually and submits the planned PV capacity to the distribution system operator (DSO). To ensure the normal operation of distribution network, the DSO needs to simulate the operation of distribution network with the planned PV capacities of customers to judge whether the distribution network can integrate the PV systems planned by customers. If the submitted PV capacities exceed the integration capability of distribution network, the capacities of customers’ PV systems need to be reduced (Armendariz et al., 2017). We assume that the DSO is responsible and capable of controlling the curtailment of the distributed PV systems to ensure the safe operation of distribution network. To determine whether the distribution network can integrate the planned PV systems, we assume that the DSO performs one-year operating simulation of the distribution network with the submitted PV capacities and estimates the curtailed PV generation. If the annual PV curtailment ratio exceeds a given threshold, the planned PV systems cannot be integrated in the distribution network and the planned PV capacities need to be reduced. For fairness, the DSO reduces the planned PV capacities in proportion to the submitted capacities of customers until the PV curtailment ratio meets the requirement. For example, the DSOs in Germany are allowed to curtail up to 3% of the annual generation of PV systems (Wiest et al., 2017), which is also set as the threshold of PV curtailment ratio in this paper. According to the installed PV capacities and the actual PV generation, the net revenues are calculated as the net present value (NPV) of customers’ PV investments. In the case studies, we assume that the load profiles of customers in the distribution network are consistent due to the lack of detailed load profile for each customer. The detailed explanations and formulations of the equipment capacity calculation of DTR are presented in STAR Methods, together with the methods of PV planning and operating simulation.

**Figure 2:**
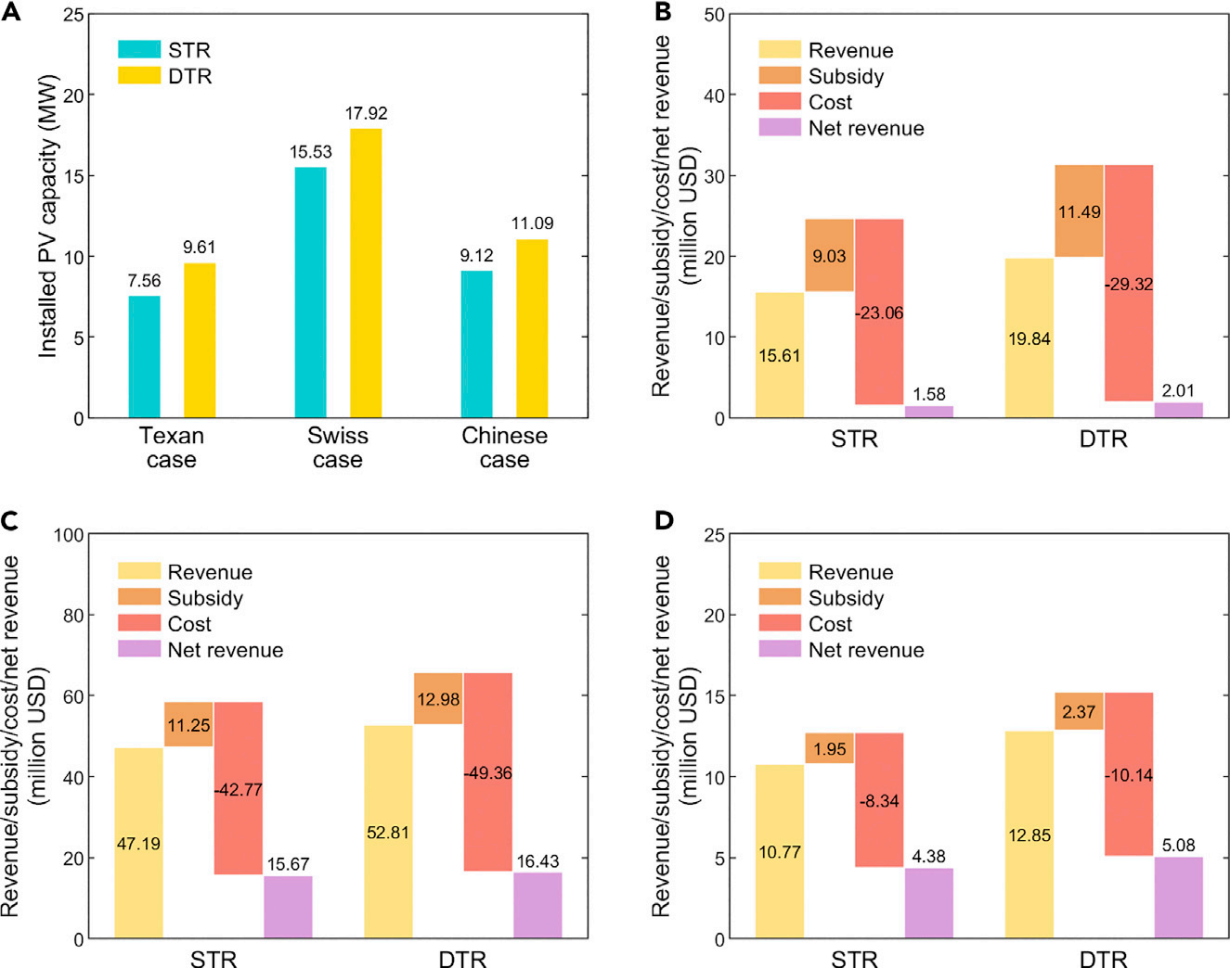
The improvements of distributed PV integration by DTR (A) The installed PV capacities of customers’ PV systems in the three distribution networks. (B) The revenue, subsidy, cost, and net revenue of customers’ PV systems in the Texan case. (C) The revenue, subsidy, cost, and net revenue of customers’ PV systems in the Swiss case. (D) The revenue, subsidy, cost, and net revenue of customers’ PV systems in the Chinese case. STR and DTR represent the results of the scenarios where STR and DTR are applied to the distribution networks, respectively.

The results of installed PV capacities and the corresponding net revenues in the two studied scenarios using 2020 weather data are shown in Figure 2. By increasing the transfer capacity of equipment to alleviate PV curtailment caused by congestion, the application of DTR enhances the integration capability of distribution networks and improves the installed PV capacities of customers. Compared to Scenario STR, the installed PV capacities in Scenario DTR increase by 27.1%, 15.4%, and 21.6% in the Texan, Swiss, and Chinese cases, respectively, as shown in Figure 2A. Although the higher installed PV capacities increase the costs, the application of DTR increases economic revenues by more, resulting in net revenue growth of 27.2%, 4.9%, and 16.0% in the Texan, Swiss, and Chinese cases, respectively. Note that we calculate the net revenues according to the NPV of distributed PV investments, while other economic indicators (e.g., benefit-cost ratio) may not be superior in Scenario DTR because the proportion of self-consumed PV generation decreases when the PV capacities increase, which reduces the revenue per unit of PV generation.

**Figure 3:**
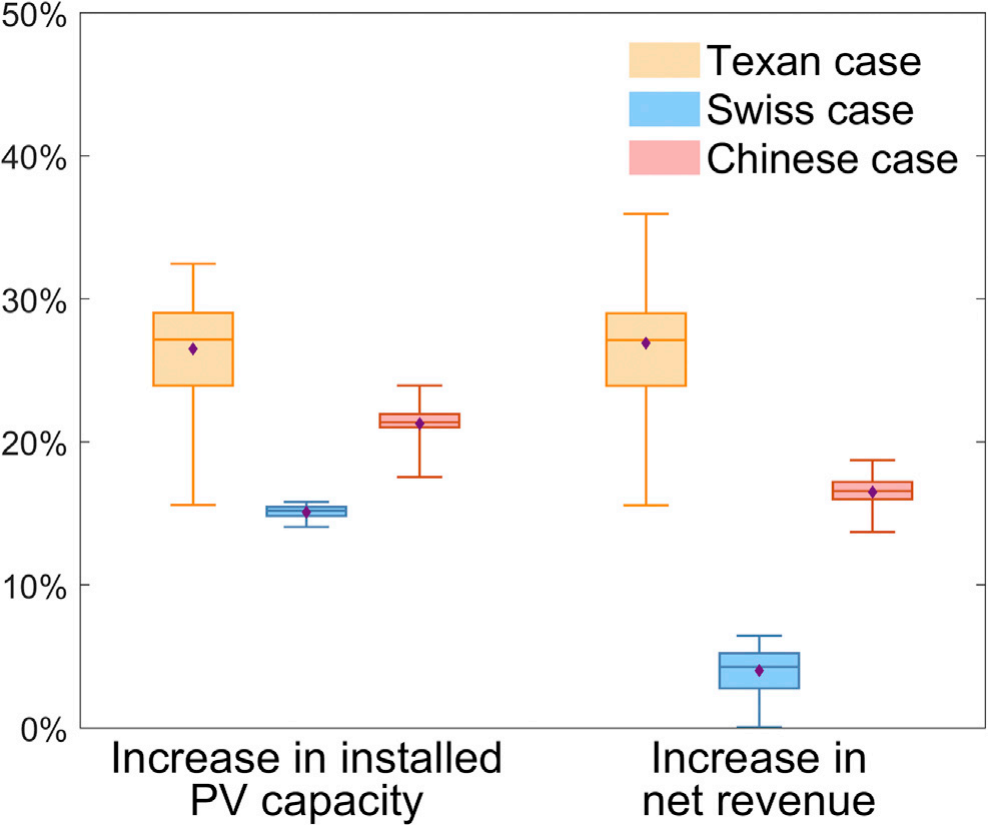
Summary of the improvements by DTR considering uncertainties The percentages of installed capacities and net revenues of customers’ PV systems increased by DTR considering the uncertainties of weather factors and PV cost, using the weather data from 2011 to 2020 and the current cost range of distributed PV systems. The boxplots represent the minimum (bottom line), first quartile (bottom of the box), median (midline in the box), third quartile (top of the box), and maximum (top line), while the purple diamonds show the average values.

We further analyze the impacts of the uncertainties of weather factors and PV cost on the improvements of distributed PV integration by DTR, using weather data from 2011 to 2020 and considering the current cost range of distributed PV systems, as shown in Figure 3. The average increases in installed PV capacities with the application of DTR in the Texan, Swiss, and Chinese cases are 26.5%, 15.1%, and 21.2%, respectively. As a result, the net revenues in the three cases increase by 26.9%, 4.0%, and 16.5% on average, respectively. In some cases with abundant solar radiation and low PV cost, the net revenue increased by DTR could reach 36.0%. Figure 3 demonstrates that of the three cases the application of DTR increases the net revenue the most in the Texan case, which is mainly due to the tariff policy for the distributed PV generation. In the Texan case, we use the value of solar (VOS) tariff (Austin Energy 2021), which is adopted by Austin Energy (the municipal utility of Austin, Texas). Residential consumers obtain a VOS credit for every kWh generated at a rate slightly lower than the residential electricity price. The VOS credit is the same for self-consumption and surplus generation fed into the grid. In contrast, the tariff for surplus PV generation is lower than the tariff for self-consumption in Switzerland and China (IEA 2020b, c). As the extra PV generation accommodated by DTR belongs to the generation fed into the grid, of the three cases, the percentage of net revenue increased by DTR is the highest in the Texan case.

### Impacts of climate change

As the application of DTR is highly dependent on weather conditions, we analyze the impacts of climate change on the improvements of distributed PV integration by DTR in this section. We estimate the percentage increases in installed PV capacities and net revenues in Scenario DTR compared to Scenario STR in 2025 and 2100 by using weather data from the CMIP6 climate model (EC-Earth Consortium 2019). We calculate the installed PV capacities and net revenues using sets of weather data from the regions of the distribution network systems, namely, the Hill Country Region of Texas (Texas Parks and Wildlife 2021), Switzerland, and Anhui Province of China. We assume that the equipment nameplate capacity in 2025 is the same as the nameplate capacity given in the distribution network systems. The equipment nameplate capacity in 2100 is calculated according to the temperature data in 2025 and 2100. As the nameplate capacity is typically calculated under the most extreme weather conditions, the nameplate capacity in 2100 is lower than the nameplate capacity in 2025 due to the increased temperature. The detailed calculation method for analyzing the impacts of climate change is presented in STAR Methods.

**Figure 4:**
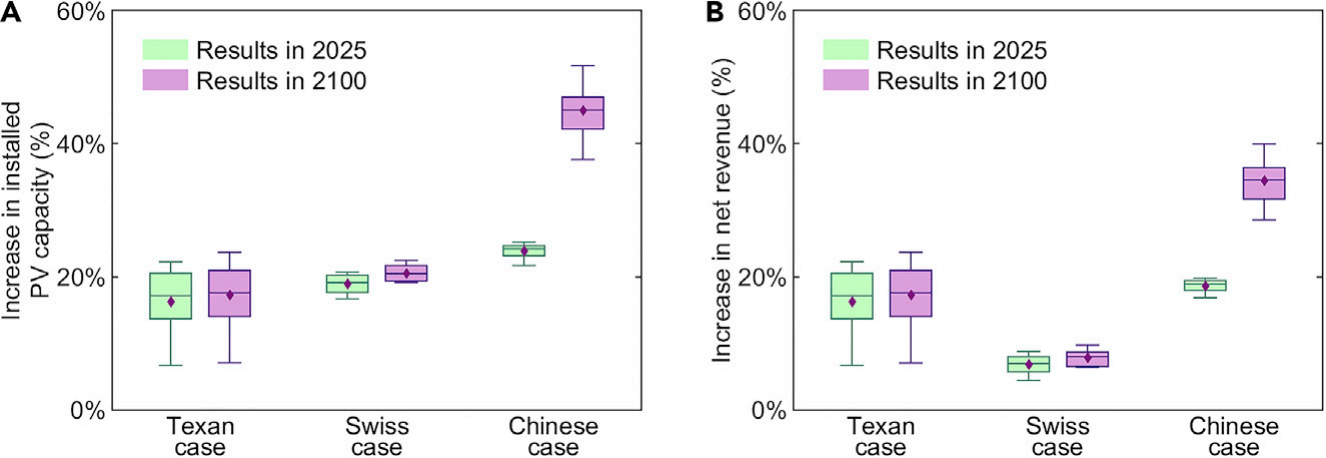
Improvements of distributed PV integration by DTR in 2025 and 2100 (A) The percentage increase in installed PV capacities in Scenario DTR compared to Scenario STR. (B) The percentage increase in net revenues in Scenario DTR compared to Scenario STR. The boxplots show the percentage increases in installed PV capacities and net revenues of customers’ PV systems in Scenario DTR compared to Scenario STR in 2025 and 2100. The simulation results are calculated using multiple sets of weather data from the Hill Country Region of Texas (Texas Parks and Wildlife 2021), Switzerland, and Anhui Province, which contain 16, 12, and 26 sets of climate model data, respectively. Each set of weather data is substituted into the simulation calculation to obtain a result, and these results are summarized in the boxplots. The boxplots represent the minimum (bottom line), first quartile (bottom of the box), median (midline in the box), third quartile (top of the box), and maximum (top line), while the purple diamonds show the average values.

Figure 4 illustrates that the projected climate change has positive impacts on the improvements of distributed PV integration by DTR in the three studied cases. As shown in Figure 4A, the percentages of installed PV capacities increased by DTR in 2025 are 16.2%, 18.9%, and 23.9% in the Texan, Swiss, and Chinese cases and increase to 17.3%, 20.6%, and 45.0% in 2100, respectively. With the higher installed PV capacities, the average percentages of net revenues increased by DTR grows with the climate change, from 16.2%, 6.8%, and 18.7% in 2025 to 17.9%, 7.8%, and 34.5% in 2100 in the Texan, Swiss, and Chinese cases, respectively, as shown in Figure 4B. The positive impacts arise from the fact that the STR of equipment, which is commonly calculated under extreme weather conditions, gradually decreases with climate change due to the increased temperature, leading to higher margins for the application of DTR to enhance equipment capacity. Relative to the Texan and Swiss cases, the percentages of the installed PV capacities and net revenues increased by DTR notably improve for the Chinese case with climate change, with average increases of 21.1% and 15.8%. The greater improvements in the Chinese case largely follow from the fact that the distribution system in the Chinese case employs overhead lines, whereas underground cables are assumed in the other two cases. Compared to underground cables, the weather conditions around overhead lines are more volatile with climate change, leading to more transfer capacity loss of STR in 2100. As a result, the application of DTR provides greater enhancements in grid transfer capacity and more significant improvements in distributed PV integration in the Chinese case.

### Impacts of PV tariff and subsidy policies

The economics of distributed PV systems is closely related to PV tariff and subsidy policies, as are the improvements of distributed PV integration by DTR. We analyze the impacts of PV tariff and subsidy policies on the improvements by DTR, assuming that the three different PV policies (Texan, Swiss, and Chinese policies) are respectively applied in the three cases. Note that we aim to analyze the improvements by DTR under different assumed PV policy mechanisms rather than practical application scenarios. Under the Texan policy, we use the PV policy adopted by Austin Energy, which is the municipal utility of Austin, Texas. Residential consumers can receive subsidies based on the installed costs and obtain a VOS credit for every kWh generated at a rate slightly lower than the residential electricity price (Austin Energy 2021). The VOS credits are priced the same for self-consumed generation and the surplus generation fed into the grid. The VOS credits can be used to offset electricity bills, but the surplus credits cannot be paid in cash. Under the Swiss policy, customers can receive subsidies proportional to the initial investment when building PV systems. The self-consumed PV generation is valued at the residential electricity price. The DSOs are obligated to purchase the surplus PV generation fed into the grid, and the price is lower than the residential electricity price (IEA 2020c). Under the Chinese policy, the PV tariff is similar to the Swiss policy, valuing the self-consumed generation at the residential electricity price and the surplus generation at the desulfurized coal benchmark price. The subsidy in the Chinese policy is related to PV generation instead of PV investment cost (IEA 2020b). Note that we assume that only the PV tariff and subsidy mechanisms are applied to the three cases, and we do not change the values of electricity prices (customer-side price or generation-side price) in each case region. The detailed settings of the PV tariff and subsidy in the three cases under different PV policies are presented in STAR Methods.

**Figure 5:**
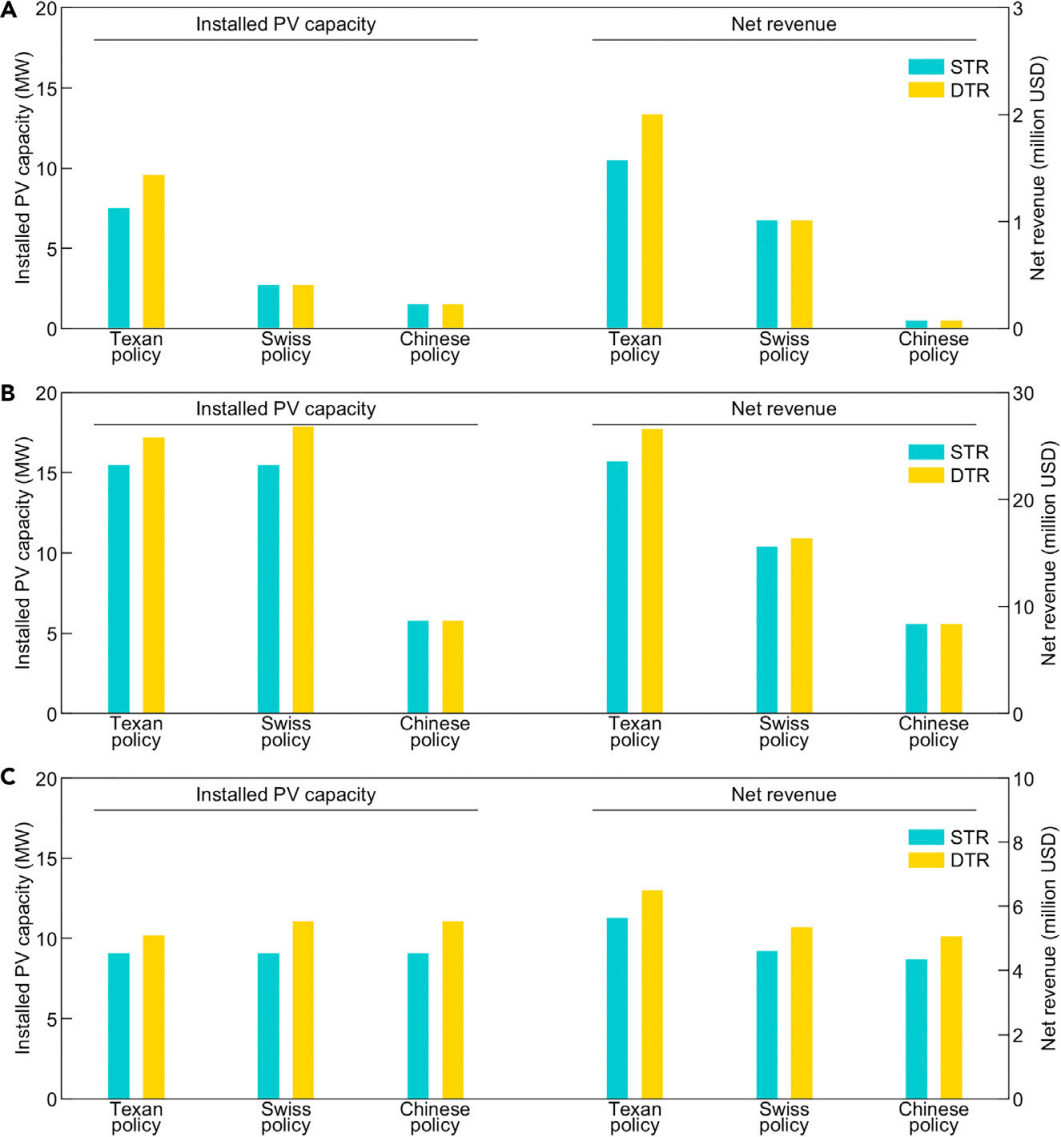
Impacts of different PV policies on the improvements by DTR (A) The installed capacities and net revenues of customers’ PV systems in the Texan case. (B) The installed capacities and net revenues of customers’ PV systems in the Swiss case. (C) The installed capacities and net revenues of customers’ PV systems in the Chinese case.

Since the application of DTR improves distributed PV integration when the PV generation exceeds the load and the surplus generation is fed into the grid, the improvements by DTR depend on the tariff for the surplus PV generation. Under the Texan policy, the VOS credits for surplus generation and self-consumed generation are the same, close to the residential electricity price. Under the Swiss and Chinese policies, the surplus generation is priced based on the generation-side price. Figure 5 shows the installed PV capacities and net revenues of each case under the three policies. As shown in Figure 5A, the application of DTR only improves the installed PV capacities and net revenues under the local policy in the Texan case. This is because that the lower prices of the surplus PV generation under the Swiss and Chinese policies reduce the planned PV capacities of customers. The existing equipment of the distribution network is sufficient to integrate the planned PV systems of customers without curtailing PV generation under the Swiss and Chinese policies. Figure 5B illustrates that the application of DTR is profitable for the distributed PV integration under the Texan policy because of the higher tariff for the surplus PV generation. However, DTR is not profitable under the Chinese policy because the generation-side price is much lower than the residential price in Switzerland, which leads to lower planned PV capacities of customers. As shown in Figure 5C, DTR effectively improves the installed PV capacities and net revenues in the Chinese case under all three policies, arising from the fact that the generation-side price is close to the residential price in China, and therefore, the prices of the surplus PV generation under different policies are all economical. In summary, these results indicate that DTR is synergistic with higher tariffs for the surplus PV generation.

### Comparison of DTR with ESSs

ESSs are believed to play a critical role in supporting high penetration of renewable energy integration in the future (IEA, 2020a; Peters et al., 2021). In this section, we compare the costs of DTR and ESSs in terms of improving distributed PV integration. Although the ESSs can provide multiple services, such as ancillary services and voltage control, we only focus on the role of the ESSs in improving the distributed PV integration in this paper. We estimate the capacities of ESSs that are required to be installed in the traditional distribution networks to integrate the PV systems of the same capacities as in Scenario DTR, using weather data in 2020 and assuming that the installed PV capacities are the same as those in Figure 2A, in Scenario DTR. Then, we estimate and compare the costs of ESSs and DTR. Since the lifetimes of different devices (e.g., ESSs and devices of DTR) are different, we calculate the annualized costs of ESSs and DTR. The methods for calculating the required capacities of ESSs and the costs of ESSs and DTR are presented in STAR Methods.

Table 1 illustrates the comparative analysis between DTR and ESSs. To integrate the PV systems of the same capacities as the application of DTR, 7.1 MWh, 8.3 MWh, and 6.1 MWh of ESSs need to be installed in the Texan, Swiss, and Chinese cases, respectively. The costs of ESSs, however, are much higher than the estimated costs of DTR, indicating that installing ESSs is not cost-competitive to enhance the integration capability of distribution networks compared to DTR.

**Table 1:** Capacity of ESSs and costs of ESSs and DTR

	Capacity of ESSs (MWh)	Annualized cost of ESSs (thousand USD)	Annualized cost of DTR (thousand USD)
Texan case	7.1	448.4	4.8
Swiss case	8.3	526.0	1.8
Chinese case	6.1	383.7	18.4

ESSs, energy storage systems; DTR, dynamic thermal rating.

## Discussion

In this work, we comprehensively analyze the improvements of distributed PV integration by DTR using distribution network systems in Texas, Switzerland, and China. The application of DTR increases the installed PV capacities by 15%–27% and improves the net revenues by 4%–27% on average in the three test cases. Using weather data from the CMIP6 climate model, we show that climate change has positive impacts on the improvements of distributed PV integration by DTR, as the STR of equipment decreases as the temperature rises. The comparative analysis of different PV policies indicates that DTR is effective under local PV policies and more profitable in the case of higher tariffs for the surplus PV generation. Moreover, DTR is more cost-competitive than deploying ESSs to integrate PV systems of the same capacities given the current cost of ESSs.

From the perspective of customers, the application of DTR can effectively integrate distributed PV systems with higher capacities and improve the profitability of distributed PV investment for customers. With the rapid development of distributed PV systems, DTR could play a more significant role in improving the integration capability of the distribution networks with high PV penetration, leading to higher benefits for customers. From the perspective of utilities, DTR could provide a solution to enhance the transfer capacity of existing equipment and improve the renewable energy integration capability of distribution networks at low cost in terms of economy, time, and land resources (Fernandez et al., 2016; Karimi et al., 2018), so as to support the goals of renewable energy development and low-carbon transition. Furthermore, it is worth noting that DTR is not a substitute for but a complement to traditional grid upgrades. It provides not only a significant enhancement in PV integration capability for existing distribution networks but also an effective strategy that can be synergized with the traditional upgrades and other technologies in future grid expansion to facilitate high renewable penetration and realize the low-carbon transformation of power grids.

At present, DTR has not been applied on a large scale in the distribution networks and has only been practically applied in several projects (Jalal 2014; Western Power Distribution 2015; Jiang et al., 2018). Nevertheless, DTR can play a more important role in supporting the future high penetration of distributed PV resources when most equipment is pushed to the brink of overload. Accurate real-time monitoring and estimation of the distribution equipment temperatures and weather conditions could facilitate the safe and reliable application of DTR. Additional weather and temperature sensors need to be installed on the original power distribution equipment. The costs of monitoring devices, however, are more cost-competitive than the ESSs analyzed in this paper and the traditional upgrades studied in the existing literature (Giannelos et al., 2018; Zhan et al., 2018). Furthermore, communication and control facilities of distribution power equipment and distributed PV systems need to be developed to process the monitoring data and to upgrade the energy management systems, which would cause extra costs. Nevertheless, with the construction of active distribution networks and the development of information and communication technologies (IRENA 2019; Chen et al., 2021), the gradually improved monitorability and controllability of devices could facilitate the application of DTR. Moreover, the devices deploying DTR can be installed only on the lines and transformers that are prone to overload, rather than all of them, which could further reduce the device cost and management complexity of deploying DTR.

The application of DTR might be limited by some factors. By treating the equipment capacity as weather-related instead of static, the application of DTR is inevitably affected by the volatility and uncertainty of weather factors due to the difficulty to predict them in advance. As the DSOs need to perform operational planning in advance to guarantee that the equipment loading does not exceed the capacity, errors in equipment capacity forecasting may affect the system reliability and lead to additional corrective action costs. Ensuring the safety and reliability of DTR is critical to increasing its attractiveness to DSOs who are accustomed to dispatching with STR. Previous research has proposed some efficient equipment capacity forecasting methods (Douglass et al., 2019) and dispatch optimization methods that can address these uncertainties such as robust programming (Qiu and Wang 2015), stochastic programming (Teng et al., 2018), and chance-constrained programming (Viafora et al., 2020). Relevant technical standards and incentivizing regulation by regulatory agencies would also favor the application of DTR.

### Limitations of the study

Our work reveals that the application of DTR could have positive improvements on distributed PV integration. Nevertheless, there are several limitations and future work can augment our analysis from the following aspects. First, we assume that the load profiles of customers in the distribution networks are consistent because only the overall load profiles of the distribution networks are available. Nevertheless, each customer’s load profile may be different from others, which would lead to different investment decisions of PV systems. Consequently, the actual application benefits of DTR may deviate from our results due to the decentralized investment decisions of customers. Second, our analysis is based on only three distribution network systems, while there are great diversities between the massive distribution networks in large regions in terms of configurations, structures, customer types, and weather conditions. The improvements of large-scale applications need to be further augmented in future work. Third, our work is focused on analyzing the application of DTR that is only capable to address the equipment overloading problem and only compare the DTR with the ESSs. Future work can augment our analysis by exploring the comparison and synergistic application of DTR with the traditional upgrades and other technologies capable of addressing the overvoltage problem, e.g., advanced inverter control and reactive power compensation. Moreover, the application cost of DTR may vary greatly by utilities, as DTR is not yet a mature and widely used technology. In the cost estimation of DTR, we only consider the costs of temperature monitoring devices. The costs of communication and control facilities need to be further investigated.

## STAR★Methods

### Key resources table

**Table:** 

REAGENT or RESOURCE	SOURCE	IDENTIFIER
**Deposited data**		

Weather data from 2011 to 2020	ECMWF	https://doi.org/10.24381/cds.e2161bac
Weather data in 2025 and 2100	EC-Earth Consortium	https://doi.org/10.22033/ESGF/CMIP6.4912
Soil characteristic parameters	Harmonized World Soil Database	http://webarchive.iiasa.ac.at/Research/LUC/External-World-soil-database/HTML/
Distribution network of Texan case	Texas A&M University Electric Grid Datasets	https://my.syncplicity.com/share/yfwu8c6lw34vkge/syn-austin-TDgrid-v03
Distribution network of Chinese case	Zu et al. (2019)	https://doi.org/10.16081/j.issn.1006-6047.2019.07.007
Data used for simulation in this paper	Li et al. (2022)	https://doi.org/10.5281/zenodo.6830724

**Software and algorithms**		

MATLAB R2020a	MathWorks	https://matlab.mathworks.com/
MATPOWER	Zimmerman et al. (2011)	https://matpower.org/
Original code in this paper	Li et al. (2022)	https://doi.org/10.5281/zenodo.6830724

### Resource availability

#### Lead contact

Further information and requests for resources should be directed to and will be fulfilled by the Lead Contact, Qixin Chen (qxchen@tsinghua.edu.cn).

#### Materials availability

This study did not generate new materials.

### Method details

#### Equipment capacity calculation method of DTR

The DTR of equipment is calculated as the highest loading that the equipment can bear without overheating key components according to the weather conditions and the thermal state of the equipment in a given time interval.

The temperature of transformers is determined by the highest winding temperature, known as the hot-spot temperature $\theta_{t}^{HS}$, which depends on the ambient air temperature $\theta_{t}^{A}$, top-oil temperature rise $\text{Δ}\theta_{t}^{TO}$ and winding hot-spot temperature rise $\text{Δ}\theta_{t}^{HS}$ (IEEE 2012):

(Equation 1) $$\theta_{t}^{HS}=\theta_{t}^{A}+\text{Δ}\theta_{t}^{TO}+\text{Δ}\theta_{t}^{HS}$$

(Equation 2) $$\text{Δ}\theta_{t}^{TO}=\left[\text{Δ}\theta_{t}^{TO,U}\left(K_{t}^{T}\right)-\text{Δ}\theta_{t-1}^{TO}\right]\left[1-e^{\frac{-\text{Δ}t}{\tau^{TO}}}\right]+\text{Δ}\theta_{t-1}^{TO}$$

(Equation 3) $$\text{Δ}\theta_{t}^{HS}=\left[\text{Δ}\theta_{t}^{HS,U}\left(K_{t}^{T}\right)-\text{Δ}\theta_{t-1}^{HS}\right]\left(1-e^{\frac{-\text{Δ}t}{\tau^{W}}}\right)+\text{Δ}\theta_{t-1}^{HS}$$

where Δt is the time interval; $\tau^{TO}$ and $\tau^{W}$ are the oil time constant and winding time constant, respectively; $\text{Δ}\theta_{t-1}^{TO}$ and $\text{Δ}\theta_{t-1}^{HS}$ are the top-oil rise and winding hot-spot rise in the last time period, respectively; and $\text{Δ}\theta_{t}^{TO,U}$ and $\text{Δ}\theta_{t}^{HS,U}$ are the ultimate top-oil rise and winding hot-spot rise, respectively, which are functions of the transformer loading ratio $K_{t}^{T}$ and represent the steady-state temperature rises if the loading remains constant indefinitely.

According to the given temperature rises in the last time period and ambient air temperature, the DTR of a transformer in time period *t* is calculated as the maximum loading without causing overheating during the time interval.

Similar to the transformers, the conductor temperature of underground cables can also be formulated as the sum of several exponential terms (Millar and Lehtonen 2006):

(Equation 4) $$\theta_{t}^{C}=\theta_{t}^{S}+\theta^{d}+\sum_{n=1}^{N}\left\{\left[A^{n}\left(I_{t}^{C},\rho_{t}^{S},\delta_{t}^{S}\right)\text{Δ}\theta_{t}^{U}\left(I_{t}^{C},\rho_{t}^{S}\right)-\text{Δ}\theta_{t-1}^{n}\right]\left[1-e^{\frac{-\text{Δ}t}{\tau_{t}^{n}\left(\rho_{t}^{S},\delta_{t}^{S}\right)}}\right]+\text{Δ}\theta_{t-1}^{n}\right\}$$

where $\theta_{t}^{C}$ is the conductor temperature; $\theta_{t}^{S}$ is the ambient soil temperature; $\theta^{d}$ is the temperature rise due to dielectric losses; $\text{Δ}\theta_{t-1}^{n}$ is the conductor temperature rise of the *n*th exponential term in the last time period; $\text{Δ}\theta_{t}^{U}$ is the ultimate conductor temperature rise over ambient except $\theta^{d}$, which is a function of cable current $I_{t}^{C}$ and soil thermal resistivity $\rho_{t}^{S}$; $A^{n}$ is the coefficient of the *n*th exponential term, which is a function of cable current $I_{t}^{C}$, soil thermal resistivity $\rho_{t}^{S}$ and soil thermal diffusivity $\delta_{t}^{S}$; and $\tau_{t}^{n}$ is the time constant of the *n*th exponential term, which is a function of soil thermal resistivity $\rho_{t}^{S}$ and soil thermal diffusivity $\delta_{t}^{S}$.

According to the given temperature rises in the last time period and weather factors (including the ambient soil temperature, soil thermal resistivity, and soil thermal diffusivity), the DTR of an underground cable is calculated as the maximum loading without causing conductor overheating in time period *t*.

The thermal model of overhead lines is formulated as the heat balance equation (IEEE 2013):

(Equation 5) $$m\cdot C_{p}\cdot \frac{d\theta_{t}}{dt}={\left(I_{t}^{O}\right)}^{2}\cdot R\left(\theta_{t}\right)+q_{t}^{s}\left(SR_{t}\right)-q_{t}^{c}\left(\theta_{t},\theta_{t}^{A},W_{t}^{S},W_{t}^{A}\right)-q_{t}^{r}\left(\theta_{t},\theta_{t}^{A}\right)$$

where *m* is the mass per unit length; $C_{p}$ is the specific heat; $I_{t}^{O}$ is the current of overhead line; $\theta_{t}$ is the conductor temperature; *R* is the conductor resistance that is related to conductor temperature $\theta_{t}$; $q_{t}^{s}$ is the solar heat gain, which is a function of solar radiation $SR_{t}$; $q_{t}^{c}$ is the convective heat loss, which is a function of conductor temperature $\theta_{t}$, ambient air temperature $\theta_{t}^{A}$, wind speed $W_{t}^{S}$ and wind angle $W_{t}^{A}$; and $q_{t}^{r}$ is the radiative heat loss, which is a function of conductor temperature $\theta_{t}$ and ambient air temperature $\theta_{t}^{A}$.

Furthermore, the thermal time constant of overhead lines is typically on the order of 5 min–20 min, implying that the conductor temperature reaches its final value in a time period of 15 min–60 min (IEEE 2013). In this paper, the dispatching time interval Δt is chosen to be 1 h. Therefore, it is reasonable to assume that the overhead lines reach thermal equilibrium in each time period, which implies that the differential term on the left-hand side of Equation (5) can be regarded as zero. Then, the DTR of an overhead line $I_{t}^{O,max}$ (maximum allowable current) can be calculated by integrating the maximum allowable temperature $\theta^{\text{max}}$ into Equation (5):

(Equation 6) $$I_{t}^{O,max}=\sqrt{\frac{q_{t}^{c}\left(\theta^{\text{max}},\theta_{t}^{A},W_{t}^{S},W_{t}^{A}\right)+q_{t}^{r}\left(\theta^{\text{max}},\theta_{t}^{A}\right)-q_{t}^{s}\left(SR_{t}\right)}{R\left(\theta^{\text{max}}\right)}}$$

Note that the above formulas for calculating DTR are presented concisely. The detailed formulas can be found in IEEE (2012), Millar and Lehtonen (2006), IEEE (2013), IEC (2015).

#### PV planning and operating methods

During the planning of the distributed PV systems, we assume that each customer in the distribution network first plans the PV capacity individually to maximize the net revenue without considering the curtailment of PV generation. To avoid excessive planning PV capacity that is certainly not accessible to the distribution network, the planned PV capacity is constrained by an upper limit set by the DSO according to the customers’ peak load. Then, the planned PV capacities of customers are submitted to the DSO. The DSO performs operating simulations of the distribution networks over the course of one year with 1-h time intervals to estimate the PV curtailment ratio. If the PV curtailment ratio exceeds the threshold, the DSO reduces the planned PV capacities in proportion to the submitted capacities of the customers until the PV curtailment ratio meets the requirement. The threshold of PV curtailment ratio is set as 3%. The calculation methods of the net revenue of the PV planning and the operating simulation of the distribution network are described in detail as follows.

The net revenue of PV investment is calculated by the NPV of the revenue, subsidy and cost:

(Equation 7) $$NR=\overset{Y}{\underset{y=0}{\sum }}\frac{1}{{\left(1+d\right)}^{y}}\left(R_{y}+S_{y}-C_{y}\right)$$

where NR is the net revenue; $R_{y}$, $S_{y}$ and $C_{y}$ are the revenue, subsidy and cost in year *y*; *Y* is the lifetime of distributed PV systems; and *d* is the discount rate.

The revenue and subsidy are calculated according to local policies. In the Texan case, we use the VOS tariff policy adopted by Austin Energy, the municipal utility of Austin, Texas (Austin Energy 2021). Residential consumers obtain a VOS credit for every kWh generated at a rate of 9.7 cents/kWh, which is slightly lower than the residential electricity price. The VOS credits can be used to offset electricity bills, but the surplus credits cannot be paid in cash. Moreover, the subsidy includes a federal solar tax credit of 26% of the installing cost from the federal government and a rebate of 2500 USD from Austin Energy. In the Swiss case, the feed-in tariff policy is adopted (IEA 2020C). The self-consumed PV generation is valued at the residential electricity price, while the tariff for the surplus PV generation fed into the grid is depended on the DSOs and is assumed to be 0.12 CHF/kWh. The PV systems are subsidized by the one-time remuneration policy, which is assumed to be 30% of the initial investment cost (IEA 2020C). In the Chinese case, the feed-in tariff policy is also adopted (IEA 2020b). The tariff for self-consumption is the residential electricity price plus 0.08 CNY/kWh subsidy, while the feed-in tariff is the desulfurized coal benchmark price plus 0.08 CNY/kWh subsidy (IEA 2020b). The specific parameters to calculate the net revenues of PV planning and investment are listed in Table S1, which are from Lazard (2020), Austin Energy (2021), U.S. Energy Information Administration (2022), Schopfer et al. (2018), Federal Electricity Commission EICom (2022), IRENA (2021), IEA (2020B, 2020C), Price Bureau of Anhui Province (2017), National Development (2020), Yan et al. (2019). To sum up, the Texan case adopts the VOS tariff, while the feed-in tariff is adopted in the Swiss and Chinese cases. The PV systems are subsidized according to the investment costs in the Texan and Swiss cases, while the subsidy is paid according to the PV generation in the Chinese case.

We assume that the revenue $R_{y}$ is the same for each year and is calculated in Equation (8) based on the local PV policies and the maximum available PV generation in one year calculated by Equations (9)-(10) (Wang et al., 2018).

(Equation 8) $$R_{y}=\left\{ \begin{array}{l} 0,\;y=0\\ R^{OY},\;y>0 \end{array}\right.$$

(Equation 9) $$P_{t}^{PV,ava}=\frac{SR_{t}}{1000}\left[1-\mu \left(\theta_{t}^{J}-25\right)\right]C^{PV}$$

(Equation 10) $$\theta_{t}^{J}=\theta_{t}^{A}+\frac{SR_{t}}{800}\left(N^{OCT}-20\right)$$

where $R^{OY}$ is the one-year revenue of PV system; $P_{t}^{PV,ava}$ is the maximum available PV output power in time period *t*; μ is the power temperature coefficient of PV system; $\theta_{t}^{J}$ is the cell temperature of PV system in time period *t*; $C^{PV}$ is the capacity of PV system; and $N^{OCT}$ is the nominal operating cell temperature of PV system.

In the Texan and Swiss cases, the subsidy is paid when the PV system is invested:

(Equation 11) $$S_{y}=\left\{ \begin{array}{l} S^{INV},\;y=0\\ 0,\;y>0 \end{array}\right.\left(\text{for Texan and Swiss cases}\right)$$

where $S^{INV}$ is the subsidy paid when the PV system is invested.

In the Chinese case, the subsidy is paid according to the PV generation. The subsidy of Chinese case is assumed to be the same for each year and is calculated by the maximum available PV generation in one year:

(Equation 12) $$S_{y}=\left\{ \begin{array}{l} 0,\;y=0\\ S^{OY},\;y>0 \end{array}\right.\left(\text{for Chinese case}\right)$$

where $S^{OY}$ is the subsidy calculated by the result of the maximum available PV generation in one year.

The cost of PV systems includes the investment cost and the operation & maintenance (O&M) cost:

(Equation 13) $$C_{y}=\left\{ \begin{array}{l} C^{INV},\;y=0\\ C^{O\&M},\;y>0 \end{array}\right.$$

where $C^{INV}$ is the investment cost of PV system; and $C^{O\&M}$ is the O&M cost of PV system.

During the PV planning of customers, the maximum available PV output power in each time period *t* is calculated according to solar radiation and ambient air temperature by Equation (9)-Equation (10). Then, the NPV with different PV capacities is calculated according to the annual PV generation. The planned PV capacity is finally determined by the PV capacity with the maximum NPV or the upper limit set by the DSO. We assume that the load profiles of customers in the distribution networks are consistent because only the overall load profiles of the distribution networks is available. Consequently, the planned PV capacity of each customer would be proportional to the customer’s peak load.

In the operating simulation of distribution networks, we calculate the PV output power in each time period through the following steps: 1) The maximum available PV output power of all PV systems is calculated according to solar radiation and ambient air temperature by Equations (9)-Equation (10). 2) The equipment capacity is set to the nameplate capacity in Scenario STR and is calculated according to the weather data and equipment temperatures in the last time period in Scenario DTR. 3) The power flow calculation is executed with the maximum available PV output power to judge whether the equipment capacity constraint and voltage constraint are satisfied. 4) If all operating constraints are satisfied, the output power of PV systems is the maximum available output power. Otherwise, the DSO needs to curtail part of PV output power to ensure the safe operation of the distribution network. For fairness, we assume that all PV systems are curtailed equally with respect to their maximum available output power (Liu et al., 2020), as shown in Equation (14). The actual PV output power is calculated by gradually increasing the curtailment ratio $\gamma_{t}$ until all operating constraints are satisfied. 5) When DTR is applied, the temperature rises of transformers and cables are calculated in preparation for calculating the equipment capacity in the next time period. In the third and fourth steps, the power flow calculation is solved by MATPOWER (Zimmerman et al., 2011).

(Equation 14) $$P_{g,t}^{PV,act}=\left(1-\gamma_{t}\right)P_{g,t}^{PV,ava},\;\forall g\in G$$

where $P_{g,t}^{PV,act}$ is the actual output power of PV system *g* in time period *t*; $\gamma_{t}$ is the curtailment ratio of PV systems in time period *t*; $P_{g,t}^{PV,ava}$ is the maximum available output power of PV system *g* in time period *t*; and *G* is the set of PV systems.

We use distribution network systems from Texas, Switzerland and China to analyze the improvements of distributed PV integration by DTR. The Texan distribution network system is selected by a three-phase balanced distribution feeder in the Combined Transmission and Distribution Synthetic Dataset (Li et al., 2020a). The Swiss distribution network system is derived from actual distribution network data. The Chinese distribution network system is taken from Zu et al. (2019). The three distribution network systems are all three-phase balanced and are suitable for simulation with MATPOWER.

The hourly weather data from 2011 to 2020 are downloaded from the ERA5-Land database (Muñoz Sabater 2019), including the air temperature, soil temperature, wind speed, wind direction, solar radiation and soil moisture content. The soil thermal resistivity and soil thermal diffusivity are calculated based on moisture content and soil characteristic parameters (Evett et al., 2012; Arkhangelskaya and Lukyashchenko 2018). The moisture content data are downloaded from the ERA5-Land database (Muñoz Sabater 2019), and the soil characteristic parameters are downloaded from the Harmonized World Soil Database (FAO/IIASA/ISRIC/ISSCAS/JRC 2012). The weather data are included in Li et al. (2022).

The net revenues of the Swiss and Chinese cases are further converted into USD for better comparison. The exchange rates of the USD to CHF and CNY are set as 0.9581 and 6.5508 respectively.

#### Impacts of climate change

We use weather data from the CMIP6 climate model (EC-Earth Consortium (EC-Earth) 2019) under the Scenario SSP5-8.5 (O’Neill et al., 2016) that updates the RCP 8.5 pathway (Moss et al., 2010) in 2025 and 2100 to estimate the impacts of climate change on the application of DTR. The climate model provides the weather data needed for simulation, except for the temperature and moisture content of the soil at the burial depth of the cables. The temperature and moisture content at the burial depth of the cables are calculated according to the temperature and moisture content at the surface soil layer and soil characteristic parameters using the soil temperature and moisture model (Evett et al., 2012; Arkhangelskaya and Lukyashchenko 2018; Diao et al., 2004; Michiorri et al., 2009; van Genuchten 1980; Schaap and van Genuchten 2006). As the climate model does not provide the weather data of exactly the same locations as the test distribution network systems due to the spatial resolution of the climate model, we use all sets of weather data that are located in the Hill Country Region of Texas (Texas Parks and Wildlife 2021), Switzerland and Anhui Province for the Texan, Swiss, and Chinese cases, respectively. The three regions contain 16, 12 and 26 sets of climate model data, respectively.

We assume that the equipment nameplate capacity in 2025 is the same as the nameplate capacity given in the distribution network systems, as 2025 is close to the present. The equipment nameplate capacity in 2100 is calculated according to the temperature data in 2025 and 2100 (IEEE 2013, 2012; IEC 2014). Then, we use each set of weather data to calculate the installed PV capacities and net revenues in Scenarios STR and DTR, respectively. The percentage increases in the installed PV capacities and net revenues in Scenario DTR compared to Scenario STR, calculated by all sets of weather data, are summarized in the boxplots, as shown in Figure 4.

#### Impacts of PV policies

To assess the impacts of different PV policies on the improvements of distributed PV integration by DTR, we recalculate the installed PV capacities and net revenues by assuming that each policy is adopted in the other two cases. When the Texan policy is applied to the Swiss and Chinese cases, the tariff for the VOS credit is converted according to the proportion of the VOS credit and residential electricity price. Moreover, the subsidy of the PV investment cost is converted according to the per-unit capacity cost of the three regions. Under the Swiss policy, self-consumed PV generation is valued at the residential electricity price. To adopt the Swiss policy to the Chinese case, the desulfurized coal benchmark price is taken as the tariff for the surplus PV generation. In the Texan case, the ratio of the tariff for the surplus PV generation to the residential electricity price is set to be the same as in the Swiss case. Furthermore, the subsidy is equal to 30% of the installed cost of the PV systems in the Texan and Chinese cases under Swiss policy. To calculate the net revenues of the Texan case and the Swiss case under the Chinese policy, the desulfurized coal benchmark price is replaced by the wholesale price. The subsidy for every kWh generated is converted according to the proportion of the subsidy tariff and residential electricity price. The electricity prices of the three case regions, including residual price, wholesale price and desulfurized coal benchmark price, are from U.S. Energy Information Administration (2022, 2021), Federal Electricity Commission EICom (2022), European Commission (2021), Price Bureau of Anhui Province (2017) and are listed in Table S2.

#### Comparison of DTR and ESSs

To approximate how many ESSs need to be installed in the traditional distribution networks to integrate PV systems of the same capacities as applying DTR, we gradually increase the capacity of ESSs and estimate the PV curtailment ratio with the installed PV capacities in Scenario DTR until the PV curtailment ratio is below the threshold. We assume that the capacity of ESS at each node in the distribution network is proportional to the installed PV capacity. How to estimate the PV curtailment ratio with ESSs is described below.

In each time period, the ESSs are set to charge if the PV output power is curtailed. The target charging power $P_{t}^{ch,tar}$ is set as the curtailed PV output power, which is equal to the difference between the maximum available PV output power $P_{t}^{PV,ava}$ and the actual PV output power without installing ESSs $P_{t}^{PV,act}$, as shown in Equation (15). Then, the charging power is constrained by the rated power $P^{rated}$ and the rated energy capacity $E^{rated}$ in Equation (16).

(Equation 15) $$P_{t}^{ch,tar}=\left\{ \begin{array}{l} P_{t}^{PV,ava}-P_{t}^{PV,act}\;\text{if}\;P_{t}^{PV,ava}>P_{t}^{PV,act}\\ 0\;\text{otherwise} \end{array}\right.$$

(Equation 16) $$P_{t}^{ch}=\text{min}\left(P_{t}^{ch,tar},P^{rated},\left(E^{rated}-E_{t-1}\right)/\eta^{ch}\right)$$

where $P_{t}^{ch}$ is the actual charging power of the ESS; $E_{t-1}$ is the state of energy of the ESS at the end of time period t−1; and $\eta^{ch}$ is the charging efficiency.

When the PV output power is less than the load, the ESSs are assumed to discharge to supply the load. The target power of discharging is equal to the load $P_{t}^{load}$ minus the maximum available PV output $P_{t}^{PV,ava}$ Equation (17). Similarly to charging, the discharging power is constrained by the rated power and the minimum allowable state of energy, as shown in Equation (18). Moreover, it is necessary to calculate the state of energy $E_{t}$ at the end of the time period before the calculation of the next time period Equation (19).

(Equation 17) $$P_{t}^{dis,tar}=\left\{ \begin{array}{l} P_{t}^{load}-P_{t}^{PV,ava}\;\text{if}\;P_{t}^{PV,ava}<P_{t}^{\text{load}}\\ 0\;\text{otherwise} \end{array}\right.$$

(Equation 18) $$P_{t}^{dis}=\text{min}\left(P_{t}^{dis,tar},P_{t}^{rated},\left(E^{t-1}-E^{min}\right)\eta^{dis}\right)$$

(Equation 19) $$E_{t}=E_{t-1}+\text{Δ}t\left(P_{t}^{ch}\eta^{ch}-P_{t}^{dis}/\eta^{dis}\right)$$

where $P_{t}^{dis}$ is the actual discharging power of the ESS; $E_{t}$ is the state of energy of ESS at the end of time period *t*; $\eta^{dis}$ is the discharging efficiency; and $E^{min}$ is the minimum allowable state of energy.

The capacity of ESSs is calculated by gradually increasing the capacity of ESS at each node in proportion to the installed PV capacity. We calculate the charging and discharging power of ESSs over the course of one year and estimate the PV curtailment ratio. We stop increasing the capacity of ESSs until the PV curtailment ratio is below the threshold and then estimate the annualized cost of ESSs. The annualized cost of ESSs includes the project cost and O&M cost. The cost parameters and lifetime of ESSs are set according to the parameters of Li-ion batteries in a recent report (Mongird et al., 2019). The per-unit capital cost, per-unit fixed O&M cost, per-unit variable O&M cost and the lifetime are set as 469 USD/kWh, 10 USD/(kWh-year), 0.0003 USD/kWh and 10 years. The charging and discharging efficiency is set as 0.92.

The annualized cost of DTR is estimated according to the costs of monitoring devices for deploying DTR. At present, the monitoring devices of overhead lines are relatively mature. Due to large variations in wind speed and wind direction, most types of devices do not measure wind speed and wind direction directly. They measure the conductor temperature and then calculate the effective wind speed according to the measured conductor temperature, ambient air temperature and solar radiation (Fernandez et al., 2016). Based on the effective wind speed, ambient air temperature and solar radiation, the transfer capacity can be calculated. The cost of each monitoring device is assumed to be 32,000 USD (World Bank Group 2016). Similarly, the environmental parameters of underground cables that need to calculate transfer capacity can be estimated by the measured cable temperature (Olsen et al., 2013). The monitoring device for underground cables is distributed temperature sensing (Olsen et al., 2013), and its cost is assumed to be 10,000 USD (Shenzhen KSD Cable 2022). For transformers, calculating transfer capacity requires monitoring ambient air temperature and equipment temperature. The cost of each monitoring device for transformers is assumed to be 4,725 USD (Advanced Power Technologies 2022). Moreover, the lifetime of monitoring devices is assumed to be 15 years (World Bank Group 2016). According to the operating simulation results of Scenario DTR, we assume that the monitoring devices are installed on the equipment whose maximum loading exceeds the nameplate capacity during the operating simulation.

## Data Availability

• This paper analyzes existing, publicly available data which are listed in the key resources table. The data used for simulation in this paper from the publicly available datasets have been deposited at Zenodo (Li et al., 2022) and are publicly available as of the date of publication. DOI is listed in the key resources table. The data of the distribution network of Swiss case will be shared by the lead contact upon request. • All original code has been deposited at Zenodo (Li et al., 2022) and is publicly available as of the date of publication. DOI is listed in the key resources table. • Any additional information required to reanalyze the data reported in this paper is available from the lead contact upon request.
