# Supplementary material for: Improving distributed PV integration with dynamic thermal rating of power distribution equipment
Source: iScience. 2022 Jul 21;25(8):104808. doi: 10.1016/j.isci.2022.104808 (PMC9385694; doi:10.1016/j.isci.2022.104808)
Supplement: Document S1. Tables S1 and S2 [file mmc1.pdf]

iScience, Volume *25*

Supplemental information

Improving distributed PV integration with dynamic thermal rating of power distribution equipment

Yinxiao Li, Yi Wang, Chongqing Kang, Jie Song, Guannan He, and Qixin Chen

Supplemental Tables

Table S1: **Parameters of PV planning and investment analysis, related to Star Methods.**

	Texan case	Swiss case	Chinese case
Per-unit investment cost	2.675 USD/W	2.313 CHF/W	5.25 CNY/W
Per-unit investment cost range	2.525-2.825 USD/W	2.113-2.513 CHF/W	5.00-5.50 CNY/W
O&M cost	1.00% of system investment cost/year		
Per-unit revenue	0.097 USD/kWh VOS credit	0.2234 CHF/kWh for self-consumption and 0.12 CHF/kWh for surplus generation	0.5653 CNY/kWh for self-consumption and 0.3844 CNY/kWh for surplus generation
Subsidy	26% of system investment cost plus 2500 USD per PV system	30% of system investment cost	0.08 CNY/kWh
PV lifetime	25 years		
Discount rate	0.05		
Power temperature coefficient	0.0045		
Nominal operating cell temperature	44 °C		

Table S2: **Electricity prices of the three cases, related to Star Methods.**

	Texas	Switzerland	China
Residual price	0.1242 USD/kWh	0.2234 CHF/kWh	0.5653 CNY/kWh
Wholesale price	0.022 USD/kWh	0.037 CHF/kWh	-
Desulfurized coal benchmark price	-	-	0.3844 CNY/kWh
